# Changrun Formula Relieves Functional Constipation by Improving Intestinal Motility in Rats

**DOI:** 10.1155/grp/5790162

**Published:** 2025-05-27

**Authors:** Qiuping Xiao, Yanqiu Hong, Xuesi Geng

**Affiliations:** ^1^Anorectal Branch, Xiamen Hospital of Traditional Chinese Medicine Affiliated to Fujian University of Traditional Chinese Medicine, Xiamen, Fujian Province, China; ^2^Dongzhimen Hospital Affiliated to Beijing University of Chinese Medicine, Beijing, China

**Keywords:** constipation, interstitial cells of Cajal, intestinal motility, Qi, traditional Chinese medicine

## Abstract

**Background and Study Aim:** Changrun Formula (CRF) is a representative traditional Chinese medicine prescription for functional constipation (FC). However, the mechanism by which CRF alleviates FC remains unclear. Therefore, this study aimed to investigate the therapeutic mechanism of CRF in an FC rat model.

**Material and Methods:** A total of 72 healthy SD rats were selected and randomly divided into six groups: the blank group, model group, hemp seed pill (HSP) group, high-dose CRF group, medium-dose CRF group, and low-dose CRF group. Except for the blank group, all the other groups were administered compound diphenoxylate via oral gavage to establish the FC rat model with impaired intestinal motility. The expression of genes related to intestinal motility in the colon tissues of rats was analyzed using Western blotting and real-time PCR. The effect of CRF on isolated colonic smooth muscle was assessed through electrophysiological analysis.

**Results:** Compared with the blank group, the other groups exhibited a longer time to expel the first black stool and a reduced number of fecal particles within 6 h, confirming the successful establishment of the FC rat model. Furthermore, the expressions of HCN1, c-kit, and SP in the colon tissue of the model group were significantly decreased, while the expression level of VIP was significantly increased. HCN1 was found to colocalize with c-kit, SP, and VIP. Treatment of CRF (high and medium doses) significantly increased the expressions of c-kit, SCF, HCN1, and HCN2, enhanced the contractile movement of colonic smooth muscle, and improved muscle tension.

**Conclusions:** CRF likely improves intestinal motility by targeting HCN1 and HCN2 ion channels and the SCF/c-kit signaling pathway, thereby alleviating FC symptoms in rats.

## 1. Introduction

Functional constipation (FC) is a functional gastrointestinal disorder characterized by persistent difficulty in defecation, prolonged defecation time, reduced stool frequency, or a sensation of incomplete evacuation, without structural abnormalities and metabolic disorders [1]. Unlike irritable bowel syndrome, FC primarily manifests as a disruption in bowel habits, significantly impacting patients' quality of life [1]. Although the exact etiology and pathogenesis of FC remain incompletely understood, they are thought to involve abnormalities in intestinal motility, the enteric nervous system, and intestinal secretion [1].

The global prevalence of FC is higher than 10% [2]. Despite various interventions, including lifestyle modifications and pharmacological treatments, patients often experience recurrent symptoms [3]. The persistent nature of FC poses significant challenges in its management, adversely impacting both the socioeconomic burden and the quality of life of affected individuals [4].

Changrun Formula (CRF), also known as Changdao Xingzhou decoction and Changrun decoction, is a classic prescription in traditional Chinese medicine (TCM) for the treatment of FC [5]. In TCM, FC is often attributed to imbalances in Yin and Qi, two fundamental concepts in TCM theory. Yin represents the nourishing, moistening, and cooling aspects of the body. A deficiency of Yin can lead to dryness in the intestines, resulting in hard stools and difficulty in defecation [6]. Qi represents the vital energy that drives bodily functions [7]. Qi stagnation or deficiency may impair the movement of the intestines, leading to sluggish bowel movements and constipation.

CRF is composed of stir-fried *Atractylodes macrocephala*, Radix Scrophulariaceae, Radix Ophiopogonis, hemp seed, *Citrus aurantium*, and betel nut. These compounds nourish Yin, benefit Qi, and promote Qi circulation, thereby moistening intestines and balancing supplementation with elimination [8]. Therefore, CRF maintains intestinal moisture while enhancing intestinal peristalsis, ultimately facilitating defecation [9].

Clinically, CRF has demonstrated significant efficacy in treating FC, improving patients' quality of life [10]. The short-term effective rate of CRF is 90.0%, with a sustained effective rate of 67.5% after 3 months of follow-up [11, 12]. Moreover, our previous clinical data indicate that CRF achieves an overall efficacy rate of 85% in FC treatment [10]. CRF has also been shown to modulate intestinal flora by increasing beneficial bacteria and reducing pathogenic bacteria, thereby optimizing the intestinal environment in FC patients [10]. Despite its clinical effectiveness, the molecular mechanisms underlying CRF's therapeutic effects on FC remain poorly understood.

Significant progress has been made in understanding gastrointestinal motility since the discovery of interstitial cells of Cajal (ICC), the pacemaker cells of the gastrointestinal tract. Ion channels, particularly hyperpolarization-activated cyclic nucleotide-gated (HCN) channels, play a critical role in generating pacemaker activity [13]. HCN channels, which are present in ICC and other spontaneously active cells such as cardiac and neuronal cells, regulate gastrointestinal pacing and represent a potential therapeutic target for colonic motility disorders [13]. In addition, the SCF/c-kit signaling pathway is essential for promoting intestinal mucus secretion, which protects the intestinal lining [14, 15], and for supporting the proliferation of ICC [16]. Nevertheless, the roles of HCN channels and the SCF/c-kit signaling pathway in FC remain underexplored.

Given that FC is associated with impaired intestinal motility and abnormal intestinal secretion [17], and CRF can enhance intestinal peristalsis and maintain intestinal hydration [10], this study aims to investigate the effects of CRF on smooth muscle movement of the isolated intestinal segments and the levels of HCN1, HCN2, SCF, and c-kit in a rat model of FC with colonic motility disorder. By exploring these mechanisms, we plan to elucidate how CRF's dual action of nourishing Yin and promoting Qi improves intestinal motility and alleviates FC symptoms.

## 2. Materials and Methods

### 2.1. Laboratory Animals

Animal studies were conducted at the Xiamen University Laboratory Animal Center. All procedures involving laboratory animals were performed in accordance with the Principles of Laboratory Animal Care (NIH no. 85-23, 1985 version) and approved by the Laboratory Animal Management and Ethics Committee of Xiamen University (approval number: XMULAC20190142).

Then, 72 healthy SD rats (36 females and 36 males) weighing 220 ± 20 g were purchased from the Xiamen University Laboratory Animal Center (License number: SYXK (Fujian) 2013-0006). The rats were housed in specific pathogen-free transparent plastic cages under controlled conditions: a 12-h light-dark cycle, temperature of 23°C, and humidity of 50%. They had free access to water and standard chow, both supplied by the Experimental Animal Center of Xiamen University.

### 2.2. Hemp Seed Pill (HSP), Compound Diphenoxylate, and CRF Preparation

HSPs were purchased from Fuzhou Neptune Golden Elephant Chinese Medicine Pharmaceutical Co., Ltd. (batch number: 1805042). Compound Diphenoxylate was purchased from Xinxiang Changle Pharmaceutical Co., Ltd. (batch number: 17040952).

CRF is a water decoction composed of 20 g fried *Atractylodes macrocephala*, 15 g Radix Scrophulariaceae, 15 g Radix Ophiopogonis, 15 g hemp seed, 15 g *Citrus aurantium*, and 15 g betel nut. Briefly, a total of 95 g of the crude drug mixture was soaked in distilled water (just covering the pieces) for 1 h, followed by reflux boiling for 30 min. The solution was filtered, and the process was repeated with fresh distilled water. The combined filtrates were centrifuged at 3500 rpm for 10 min, and the supernatant was concentrated using rotary evaporation to 47.5 mL, yielding a 200% CRF solution. For experiments, CRF decoctions containing 0.5 g/mL, 1 g/mL, or 2 g/mL of raw drug were prepared.

### 2.3. Grouping and Modeling

After a 2-week acclimatization period, the rats were randomly divided into six groups (*n* = 12 per group): the blank group, model group, HSP group, high-dose CRF group, medium-dose CRF group, and low-dose CRF group. The FC rat model was established as previously described [18]. The blank group received distilled water (2.2 mL/time, twice a day) by oral gavage, while the other groups were administered compound diphenoxylate (10 mg/kg/day) by oral gavage for 14 consecutive days. Then, 30 min after the final administration, 0.2 mL/20 g of ink was administered by gavage. The time to discharge the first black stool, the number of stools within 6 h, and stool characteristics were recorded. Rat gavage apparatuses (model: HL-GW-16-10) were purchased from Beijing Heli Kechuang Technology Development Co., Ltd.

### 2.4. Drug Administration

Following successful modeling, all rats were given distilled water (1 mL/100 g/day, twice daily) by gavage. Afterwards, the HSP group received (HSP 0.3 g/kg/d, twice daily), while the low-dose, medium-dose, and high-dose CRF groups were administered CRF at 5.5 g/kg/day, 11 g/kg/day, and 22 g/kg/day, respectively. Dosages were calculated based on adult human equivalents using the “*Methodology of Experimental Pharmacology of Traditional Chinese Medicine*”. After 14 days of treatment, rats were euthanized via intravenous injection of 150 mg/kg pentobarbital sodium. Death was confirmed by the absence of corneal reflexes, breathing, or heartbeats for over 5 min. Tissues from the ascending, transverse, and descending colon were collected for further analysis.

### 2.5. Preparation and Treatment of Smooth Muscle Strips

Smooth muscle strip preparation was performed according to a previous publication [19]. Colon tissues from the blank group were placed in precooled, oxygenated Krebs solution, and the mesentery was removed. The intestinal cavity was cut longitudinally along the mesentery, washed with Krebs solution, and fixed on a wax board, with the smooth muscle serosal layer facing upward. Muscle strips (3 mm × 10 mm) were cut along the longitudinal smooth muscle fibers for electrophysiological testing.

The muscle strips were divided into four groups: the control group, high CRF group, medium CRF group, and low CRF group (*n* = 3 per group). For electrophysiological recording, muscle strips were initially treated with 1-mL Krebs solution, and the electrophysiological curve was recorded for 3 min. After that, the control group received 1-mL Krebs solution, while the high, medium, and low CRF groups received 1 mL of 2 g/mL, 1 g/mL, and 0.5 g/mL CRF, respectively, and the electrophysiological curve was recorded for 3 min.

### 2.6. Real-Time PCR (RT-PCR)

The total RNA was extracted using an RNA extraction kit (Promega, Catalog number: LS1040) and then reverse-transcribed into cDNA using a reverse transcription kit (Promega, Catalog number: A5001). RT-PCR was performed using RT-PCR reagents (TransGen Biotech, Catalog number: AQ101-03). The thermal cycle parameters are as follows: 95°C for 30 s, 1 cycle and 95°C for 5 s, 60°C for 30s, 45 cycles. The RT-PCR primers designed by ABI Primer Express10 software are listed in Table 1 and were synthesized by Xiamen Bright Biotech Co., Ltd. The *β*-actin gene serves as the internal reference, and data were analyzed using CFX96 Manager software. The relative expression of target genes was calculated using the 2^−*ΔΔ*Ct^ method.

### 2.7. Western Blotting

Proteins were extracted using ice-cold RIPA buffer (Beyotime, Catalog number: P0013C) and quantified using BCA protein assay kit (Beyotime, Catalog number: P0010S). Denatured protein samples were separated by 12% SDS-PAGE and then transferred to PVDF membranes (Membrane Solutions, Catalog number: R7EA3809G). After incubation with primary antibody and horseradish peroxidase–conjugated secondary antibody solutions, the signals were visualized using ECL chemiluminescence. Band intensities were analyzed using ImageJ software (NIH). The primary and secondary antibody information is provided in Table 2.

### 2.8. Immunofluorescence (IF)

Colon tissues were fixed, dehydrated, and embedded in paraffin to prepare sections 4 *μ*m. Antigen retrieval was performed in 0.01 M citrate buffer (pH 6.0). Subsequently, the sections were incubated with HCN1 antibody (1:250; ab229340, Abcam), c-kit antibody (1:100; sc-365504, Santa Cruz Biotechnology), substance P (SP) antibody (1:100; sc-133143, Santa Cruz Biotechnology), and vasoactive intestinal peptide (VIP) antibody (1:100; sc-25347, Santa Cruz Biotechnology), followed by secondary antibodies (Alexa Fluor 647 Tyramide SuperBoost Kit goat anti-rabbit IgG, #B40926, Invitrogen; CoraLite488-conjugated Affinipure Goat Anti-Mouse IgG, SA00013-1, Proteintech). Finally, the DNA was stained with DAPI, and images were captured using a Zeiss LSM-510 laser scanning fluorescence microscope. Fluorescence intensity was quantified using ImageJ software.

### 2.9. Statistical Analysis

Data were analyzed using SPSS 22.0 software and expressed as mean ± standard error of the mean (SEM). For normally distributed data with homogeneous variance, one-way ANOVA followed by LSD or SNK tests was used. For nonhomogeneous variances, Dunnett's T3 test was applied. Nonnormally distributed data were analyzed using the rank sum test. A *p* value < 0.05 was considered statistically significant.

## 3. Results

### 3.1. The FC Rat Model Was Successfully Established

To investigate the role of CRF in regulating intestinal smooth muscle movement, we first established an FC rat model. During the experiment, no rats died, indicating that the FC model prepared by the compound diphenoxylate method is safe and stable. The model group, HSP group, high-dose CRF group, medium-dose CRF group, and low-dose CRF group have statistically significant differences in the time to discharge the first black stool, the number of fecal particles discharged in 6 h, and the weight of feces compared with Blank group (*p* < 0.05), indicating that the FC rat model was successfully established (Table 3).

### 3.2. HCN1, c-Kit, SP, and VIP Expressions Were Altered in the Colon of FC Rats

**Figure 1:**
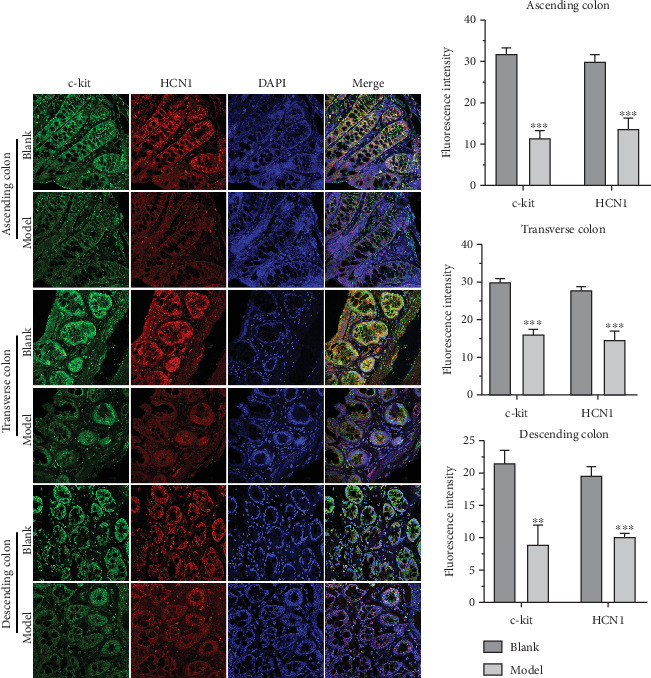
HCN1 and c-kit in the blank and model groups were detected by IFA. (a) The expression levels of HCN1 and c-kit in the colon of the blank group and model group were detected by IFA. (b) Histogram of fluorescence intensity of HCN1 and c-kit. ⁣^∗∗^*p* < 0.01, ⁣^∗∗∗^*p* < 0.001 vs. blank.

**Figure 2:**
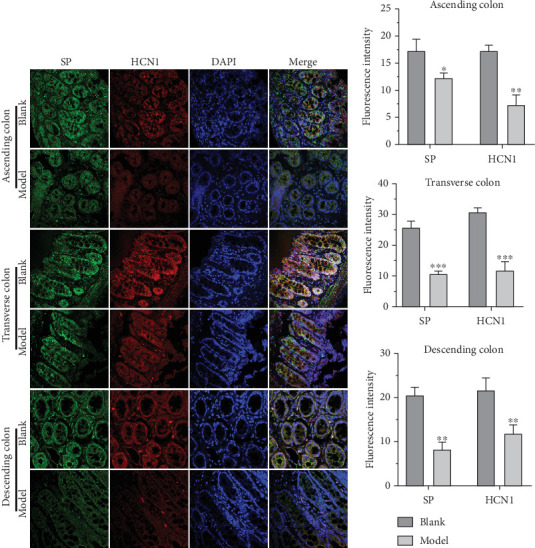
HCN1 and SP in the blank and model groups were detected by IFA. (a) The expression levels of HCN1 and SP in the colon of the blank group and model group were detected by IFA. (b) Histogram of fluorescence intensity of HCN1 and SP. ⁣^∗^*p* < 0.05, ⁣^∗∗^*p* < 0.01, ⁣^∗∗∗^*p* < 0.001 vs. blank.

**Figure 3:**
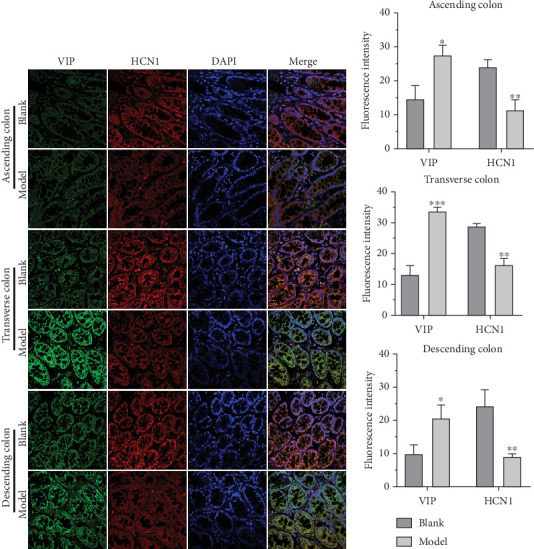
HCN1 and VIP in the blank and model groups were detected by IFA. (a) The expression levels of HCN1 and VIP in the colon of the blank group and model group were detected by IFA. (b) Histogram of fluorescence intensity of HCN1 and VIP. ⁣^∗^*p* < 0.05, ⁣^∗∗^*p* < 0.01, ⁣^∗∗∗^*p* < 0.001 vs. blank.

Given that the expressions of c-kit, SP, and VIP are frequently dysregulated in [20, 21] and HCN channels may serve as therapeutic targets for colonic motility disorders [22], we then assessed their expression in the colon of FC rats by IFA. The results revealed that c-kit, HCN1, and SP levels were significantly decreased, while VIP expression was upregulated in various regions of the colon in FC rats compared to the blank group (Figures 1, 2, and 3). These findings suggest that colonic pacemaking potentials and neurotransmitter production are impaired in FC rats, which may underlie the defective colonic motility observed in these animals.

### 3.3. CRF Rescued the Expressions of c-Kit, SCF, HCN1, and HCN2 in the Colon of FC Rats

**Figure 4:**
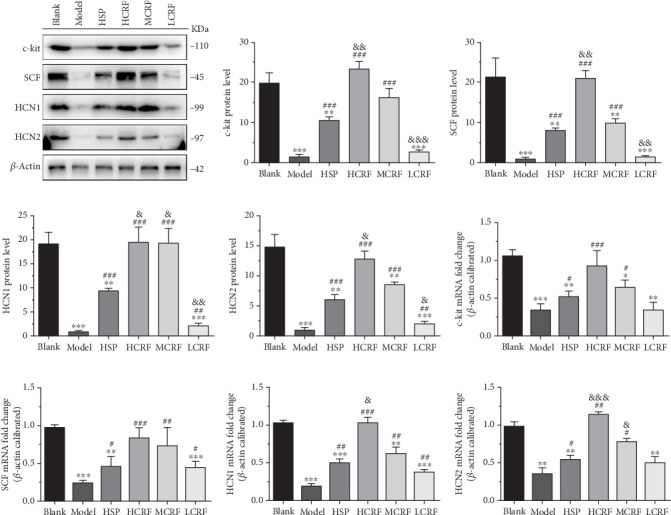
The expressions of c-kit, SCF, HCN1, and HCN2 mRNA and protein in the ascending colon of rats. (a) The protein levels of c-kit, SCF, HCN1, and HCN2 in the ascending colon of FC rats were detected by Western blotting. The relative quantitative histogram of (b) c-kit, (c) SCF, (d) HCN1, and (e) HCN2 protein levels in the ascending colon of FC rats. The mRNA levels of (f) c-kit, (j) SCF, (h) HCN1, and (i) HCN2 in the ascending colon of rats were detected by RT-PCR. HSP: hemp seed pill group; HCRF: high-dose CRF group; MCRF: medium-dose CRF group; LCRF: low-dose CRF group. ⁣^∗^*p* < 0.05, ⁣^∗∗^*p* < 0.01, ⁣^∗∗∗^*p* < 0.001 vs. blank; ^#^*p* < 0.05, ^##^*p* < 0.01, ^###^*p* < 0.001 vs. model; and ^&^*p* < 0.05, ^&&^*p* < 0.01, ^&&&^*p* < 0.001 vs. HSP.

**Figure 5:**
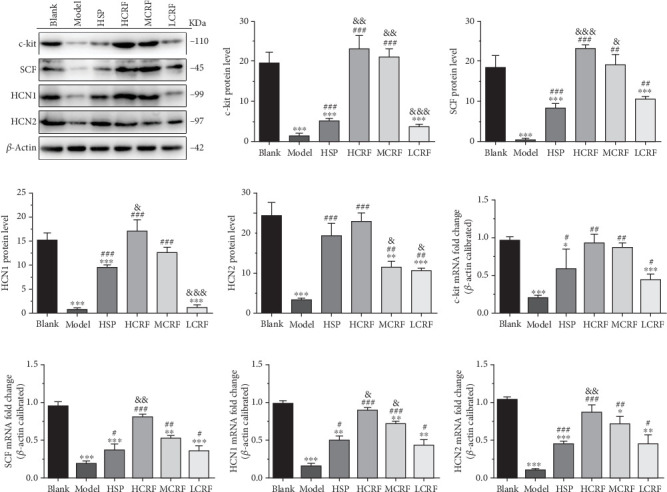
The expressions of c-kit, SCF, HCN1, and HCN2 mRNA and protein in the transverse colon of rats. (a) The protein levels of c-kit, SCF, HCN1, and HCN2 in the transverse colon of rats were detected by Western blotting. The relative quantitative histogram of (b) c-kit, (c) SCF, (d) HCN1, and (e) HCN2 protein levels in the transverse colon of rats. The mRNA levels of (f) c-kit, (j) SCF, (h) HCN1, and (i) HCN2 in the transverse colon of rats were detected by RT-PCR. HSP: hemp seed pill group; HCRF: high-dose CRF group; MCRF: medium-dose CRF group; LCRF: low-dose CRF group. ⁣^∗^*p* < 0.05, ⁣^∗∗^*p* < 0.01, ⁣^∗∗∗^*p* < 0.001 vs. blank; ^#^*p* < 0.05, ^##^*p* < 0.01, ^###^*p* < 0.001 vs. model; and ^&^*p* < 0.05, ^&&^*p* < 0.01, ^&&&^*p* < 0.001 vs. HSP.

**Figure 6:**
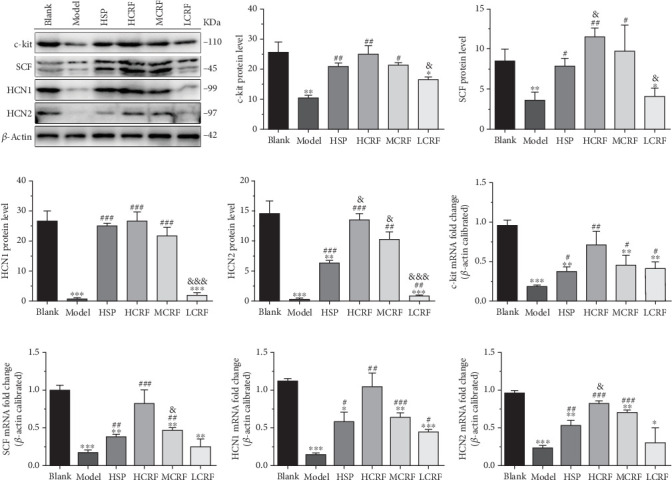
The expressions of c-kit, SCF, HCN1, and HCN2 mRNA and protein in the descending colon of rats. (a) The protein levels of c-kit, SCF, HCN1, and HCN2 in the descending colon of rats were detected by western blotting. The relative quantitative histogram of (b) c-kit, (c) SCF, (d) HCN1, and (e) HCN2 protein levels in the descending colon of rats. The mRNA levels of (f) c-kit, (j) SCF, (h) HCN1, and (i) HCN2 in the descending colon of rats were detected by RT-PCR. HSP: hemp seed pill group; HCRF: high-dose CRF group; MCRF: medium-dose CRF group; LCRF: low-dose CRF group. ⁣^∗^*p* < 0.05, ⁣^∗∗^*p* < 0.01, ⁣^∗∗∗^*p* < 0.001 vs. blank; ^#^*p* < 0.05, ^##^*p* < 0.01, ^###^*p* < 0.001 vs. model; and ^&^*p* < 0.05, ^&&^*p* < 0.01, ^&&&^*p* < 0.001 vs. HSP.

To determine whether CRF treatment can restore the disrupted c-kit pathway and HCN channels in the colon of FC rats, we evaluated the expressions of c-kit, SCF, HCN1, and HCN2. Western blotting and RT-PCR revealed that compared with the blank group, the expressions of c-kit, SCF, HCN1, and HCN2 in the ascending, transverse, and descending colon of the model group were significantly reduced. However, the expression of these genes was markedly improved in the HSP group and CRF-treated groups (Figures 4, 5, and 6). Notably, the high- and medium-dose CRF were more effective than HSP in upregulating the expression of these genes, while the low-dose CRF showed less pronounced effects (Figures 4, 5, and 6). These results demonstrate that CRF dose dependently restores the colonic expression of these genes in FC rats, highlighting its therapeutic potential in addressing colonic motility disorders.

### 3.4. CRF Promotes the Contraction of Isolated Colonic Smooth Muscle of FC Rats

**Figure 7:**
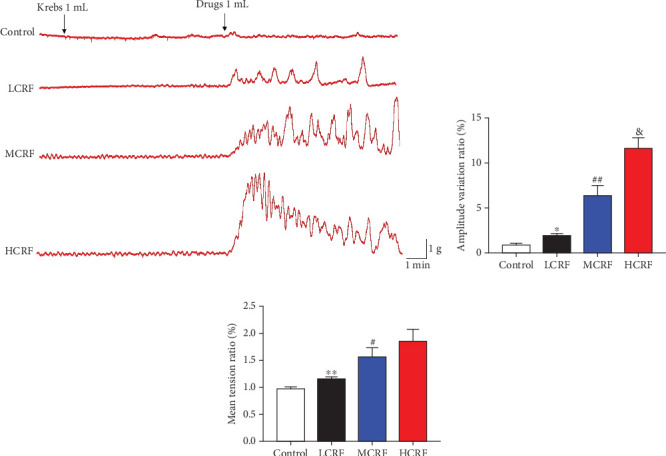
CRF promotes the contraction of the isolated colonic smooth muscle of FC rats. (a) After treating the isolated colonic smooth muscle strips with different concentrations of CRF, the electrophysiological changes of the muscle strips were detected. (b) A quantitative comparison chart of the amplitude change rate of the effect of CRF on rat colonic smooth muscle strips. (c) Quantitative comparison chart of the average tension change rate of the effect of CRF on rat colonic smooth muscle strips. HCRF: high-dose CRF group; MCRF: medium-dose CRF group; LCRF: low-dose CRF group. ⁣^∗^*p* < 0.05, ⁣^∗∗^*p* < 0.01 vs. control; ^#^*p* < 0.05, ^##^*p* < 0.01 vs. LCRF; and ^&^*p* < 0.05 vs. MCRF.

To directly assess the effects of CRF on colonic smooth muscle contraction in FC rats, we performed electrophysiological assays. The results demonstrated that, compared to the control group, CRF significantly enhanced the contractile activity of colonic smooth muscle and improved muscle tension, with more pronounced effects observed at higher doses (Figure 7). These findings indicate that CRF effectively enhances colonic smooth muscle contraction in FC rats, further supporting its therapeutic potential for improving colonic motility.

## 4. Discussion

In this study, we explored the effects of CRF on colonic smooth muscle motility and the expression of genes encoding pacemaking channels and neurotransmitters in an FC rat model. Our findings provide strong evidence that CRF enhances colonic smooth muscle contraction and restores the expression of key regulators of intestinal motility.

Mammalian HCN channels comprise four subtypes (HCN1–HCN4), with HCN1 and HCN2 being the most abundant in the gastrointestinal tract [12, 13, 23]. Studies have shown that HCN1 is specifically distributed in ICC, particularly the ICC-MY located between the circular and longitudinal muscles, while HCN2 is distributed on nerve fibers and coexists with neurotransmitters [11, 12, 24]. The impaired expression of HCN2 protein leads to impaired gastrointestinal motility in mice [25]. These findings suggest that HCNs, especially HCN1 and HCN2, play an important role in regulating gastrointestinal motility and may be implicated in the pathology of FC.

Supporting this theory, our study showed that the protein and mRNA levels of HCN1 and HCN2 in the ascending colon, transverse colon, and descending colon of the model group were lower than those in the blank group. Treatment with high- or medium-dose CRF significantly increased the protein and mRNA levels of HCN1 and HCN2 in FC rats, suggesting CRF may alleviate FC symptoms by restoring the expression of these proteins. Nevertheless, whether CRF affects the HCN3 and HCN4 expressions and the precise mechanisms by which CRF regulates HCN1 and HCN2 require further investigation.

The colonic mucus layer plays a vital role in protecting intestinal epithelial cells from symbiotic bacteria and pathogens, as well as lubricating the colon [26, 27]. The SCF/c-kit signaling pathway is known to promote mucus secretion in the colon [15]. In our study, the expression of c-kit and SCF in the colon tissue of FC rats was significantly decreased, indicating that the occurrence of FC is related to the abnormal expression of c-kit and SCF and the disruption of the SCF/c-kit signaling pathway. Notably, the levels of c-kit and SCF were positively correlated with the dose of CRF, suggesting that CRF treated FC by upregulating c-kit and SCF expressions, thereby repairing the SCF/c-kit signaling pathway, enhancing mucus secretion, protecting the intestinal tract from pathogens, and improving colonic lubrication. However, the mechanism underlying CRF's regulation on the c-kit and SCF expressions in the colon of FC rats warrants further exploration.

It is important to note that our conclusions are based solely on an FC rat model. Whether CRF exerts similar effects in other FC models or in human FC patients requires further investigation.

## 5. Conclusions

In summary, our study demonstrates that CRF improves the mRNA and protein levels of HCN1, HCN2, SCF, and c-kit in the colon tissues of FC rats, providing preliminary insights into the molecular mechanisms underlying CRF's therapeutic effects on FC. These findings offer a theoretical and experimental foundation for the clinical application of CRF in FC treatment and highlight its potential for expanding the prevention and treatment of FC within the framework of TCM.

## Figures and Tables

**Table 1:** Primers for RT-PCR.

**Name**	**Sequence (5**⁣′**-3**⁣′**)**	**Gene ID**
*β*-actin- F	CTGGCTCCTAGCACCATGAA	81822
*β*-actin- R	AAAACGCAGCTCAGTAACAGTC	
c-kit-F	GACAGTTGCCGTGAAGATGC	64030
c-kit-R	GCCAAGCAGGTTCACAATATTCA	
SCF-F	TCAAGGACTTCATGGTGGCAT	60427
SCF-R	ATTCCTAAGGGAACTGGCTGC	
HCN1-F	CAGAGCACTTCGGATCGTGA	84390
HCN1-R	GGAGCAGCATCATGCCAATG	
HCN2-F	ACTTCCGCCAGAAGATCCAC	114244
HCN2-R	AGCTTCCGGCAGTTGAAGTT	

Abbreviations: F, forward primer; R, reverse primer.

**Table 2:** Antibodies for Western blotting.

**Classification**	**Name of antibody**	**Manufacturer**	**Catalog no.**	**Dilution**
Primary antibody	c-kit	Abcam	ab256345	1:1000
	SCF	Proteintech	26582-1-AP	1:1000
	HCN1	Abcam	ab84817	1:750
	HCN2	Abcam	ab84816	1:500
	*β*-actin	Abcam	ab8226	1:1000
	GAPDH	Proteintech	60004-1-Ig	1:20,000

Secondary antibody	HRP-conjugated Affinipure Goat Anti-Rabbit IgG	Abcam	ab205718	1:3000
	HRP-conjugated Affinipure Goat Anti-Mouse IgG	Abcam	ab6721	

**Table 3:** Comparison of defecation of 6 groups of rats.

**Group name**	**Number**	**Time to first bowel movement (min)**	**Number of defecation particles**	**Stool weight (g)**
Blank group	12	82.67 ± 8.28	12.50 ± 1.69	2.62 ± 0.46
Model group	12	178.58 ± 17.40^a^	6.25 ± 0.89^a^	1.28 ± 0.23^a^
HSP group	12	176.67 ± 24.49^a^	6.33 ± 1.20^a^	1.18 ± 0.25^a^
HCRF group	12	153.42 ± 21.56^a^	6.17 ± 1.03^a^	1.40 ± 0.29^a^
MCRF group	12	191.50 ± 19.72^a^	5.42 ± 1.34^a^	1.24 ± 0.36^a^
LCRF group	12	156.58 ± 17.66^a^	6.00 ± 0.807^a^	1.48 ± 0.21^a^

Abbreviations: HCRF, high-dose CRF; HSP, hemp seed pill; LCRF, low-dose CRF; MCRF, medium-dose CRF.

^a^ *p* < 0.05 vs. blank group.

## Data Availability

All materials and data in the present study are available from the corresponding author on reasonable request.
